# Hip stability parameters with dual mobility, modular dual mobility and fixed bearing in total hip arthroplasty: an analytical evaluation

**DOI:** 10.1186/s12891-022-05280-2

**Published:** 2022-04-20

**Authors:** Domenico Tigani, Lorenzo Banci, Riccardo Valtorta, Luca Amendola

**Affiliations:** 1 Department of Orthopaedics, Maggiore Hospital, Largo Nigrisoli 2, 40100 Bologna, Italy; 2 Department of Clinical Research and D&R, Permedica S.P.A, Via Como 38, 23807 Merate, Italy

**Keywords:** Dual mobility, Modular dual mobility, Jumping distance, Oscillation angle, Total hip arthroplasty, Dislocation

## Abstract

**Background:**

Use of dual mobility (DM) in total hip arthroplasty has gained popularity due to the ability to reduce dislocation through increased jumping distance and impingement-free arc of movement. Recently, modular dual mobility (modDM) systems were introduced to give the possibility to use DM with standard metal-backed shells, however few has been studied to date regarding how jumping distance and the center of rotation change with modDM. The objective of this study was to evaluate, through analytical simulation, how jumping distance, center of rotation and arc of movement change between DM and standard cups with modDM or fixed bearings (FB).

**Methods:**

3D-models of DM and standard press-fit cups with modDM or FB liners were used to simulate DM, modDM and FB implant configurations, matched for same cup size, according to same cup position and different femoral head diameters. Jumping distance was calculated and center of rotation lateralization and oscillation angles were measured for each size of these three implant configurations.

**Results:**

Jumping distance with modDM was reduced by -3.9 mm to -8.6 mm in comparison with DM, from 48 to 64 mm size, but resulted comparable to polyethylene 36 mm FB and increased by + 1.1 mm and + 1.4 mm than ceramic 36 and 40 mm FBs for sizes > 54 mm. ModDM lateralized the center of rotation up to + 2.5 mm and + 4.0 mm in comparison with DM and FBs, respectively. Oscillation angle with modDM resulted higher than + 16°, + 23°, + 17° and + 14° in comparison to DM, 28 mm, 32 mm and 36 mm FB cups, respectively, for 56 mm cup size.

**Conclusions:**

According to its specific design, modDM might change hip stability parameters in comparison to DM, worsening jumping distance and center of rotation position, but increasing arc of movement. As not restoring stability parameters in the same fashion, modDM implants should be properly used when DM cups are not feasible.

## Background

Instability after total hip arthroplasty (THA) continues to be one of the leading causes of early revision and the first reason for failure after revision THA [1].

Risk factors for early dislocation include patient-related and surgical-related factors as well as factors linked to the implant. Although multifactorial, it is well accepted that stability in THA improves with larger-sized femoral heads [2, 3]. Reasons supporting use of larger femoral heads are primarily the ability to provide a wider impingement-free arc of movement and increased jumping distance (JD) [4, 5].

Dual mobility (DM) concept was developed and introduced in THA in 1974 in order to reduce postoperative hip instability [6, 7]. The idea of a dual articulation was to combine Charnley’s low friction principle to reduce polyethylene wear by small diameter femoral heads [8] with the Mckee–Farrar concept of using larger diameter femoral heads to enhance hip stability [9]. DM has been reported to be effective in decreasing the risk of postoperative instability both in primary and revision THA [10].

In the last decade the introduction of a modular DM (modDM) metal inlay allowed the use of a DM polyethylene liner with a press-fit metal-backed shell for an “hybrid” modDM THA in high-risk patients for hip dislocation.

The modDM option is nowadays available on the market for many acetabular cup systems as Stryker MDM, Smith&Nephew OR3O with Oxinium® technology, Zimmer-Biomet G7, DePuy Pinnacle, Lima Delta TT, Corin Trinity and to date, modDM acetabular systems are providing excellent results with rare dislocations after revision THA [11, 12]. Recently, several studies are reporting on metal release caused by the modularity of DM inlay, showing acceptable blood metal levels [13–15]. However, few data has been published so far regarding biomechanical differences in terms of JD and prosthetic ROM between conventional DM and modDM systems [16].

The objective of this study was to evaluate, through an analytical 3D-modelling simulation, how JD, center of rotation (CR) position and prosthetic arc of movement change in consideration of DM, modDM and fixed bearing (FB) cups, matched for same cup size, according to same cup position, different head diameters and femoral head offset.

## Methods

### Definition of the parameters

A Cartesian reference landmark was defined: O was the center of the cup, Oz was the cranio-caudal axis, Oy was the lateral-medial axis, and Ox the posterior-anterior axis.

**Fig. 1:**
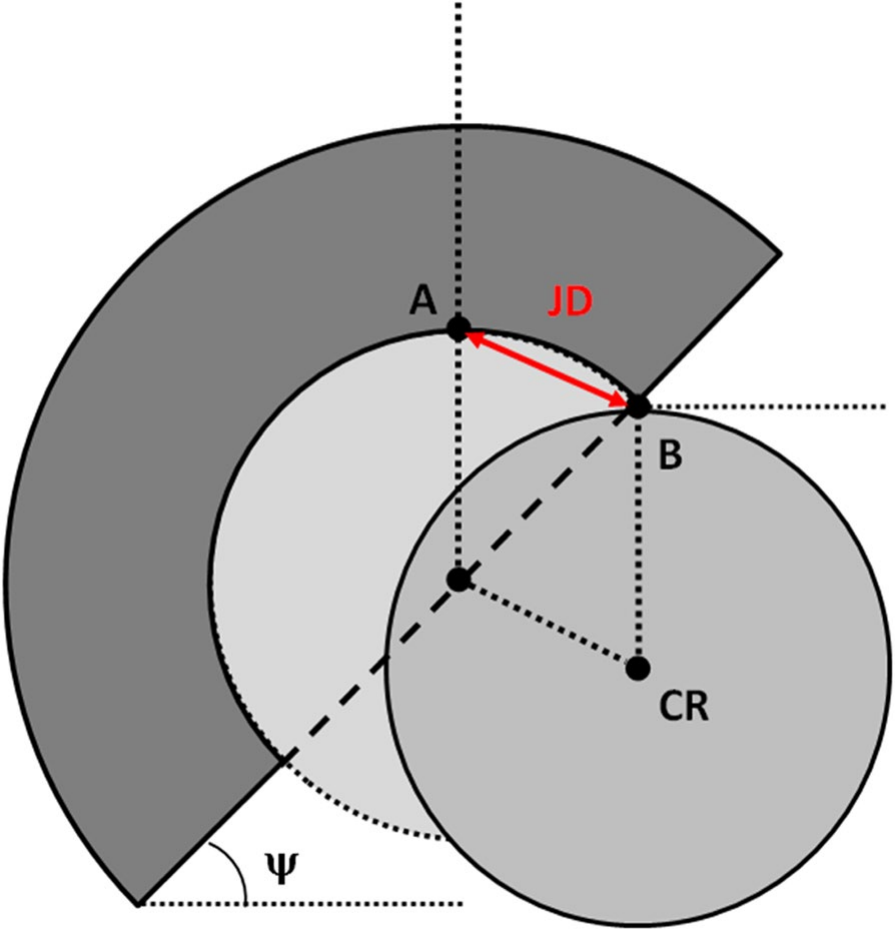
Jumping distance (JD) is defined as the lateral translation distance AB of the femoral head center (CR) required for a femoral head to dislocate from an acetabular socket

JD is defined as the lateral translation distance of the femoral head center (CR) required for a head to dislocate from a socket (Fig. 1).

We used the formula by Sariali [17], reported below, to calculated JD, which is a function of 4 variables: α, β, R, offset.

$$JD=2Rsin\left[(\pi/\mathit2-\Psi-arcsin\ \left(offset/R)\right)/2\right]$$  

where:

Ψ is the planar cup inclination angle measured on the frontal plane by using the following formula and corresponds to the projection of the abduction angle (α) on the frontal plane.

Ψ = arctan [tan(α) × cos(β)].

α is the cup abduction angle.

β is the cup anteversion angle on the cross-sectional plane.

*R* is the radius of the femoral head.

**Fig. 2:**
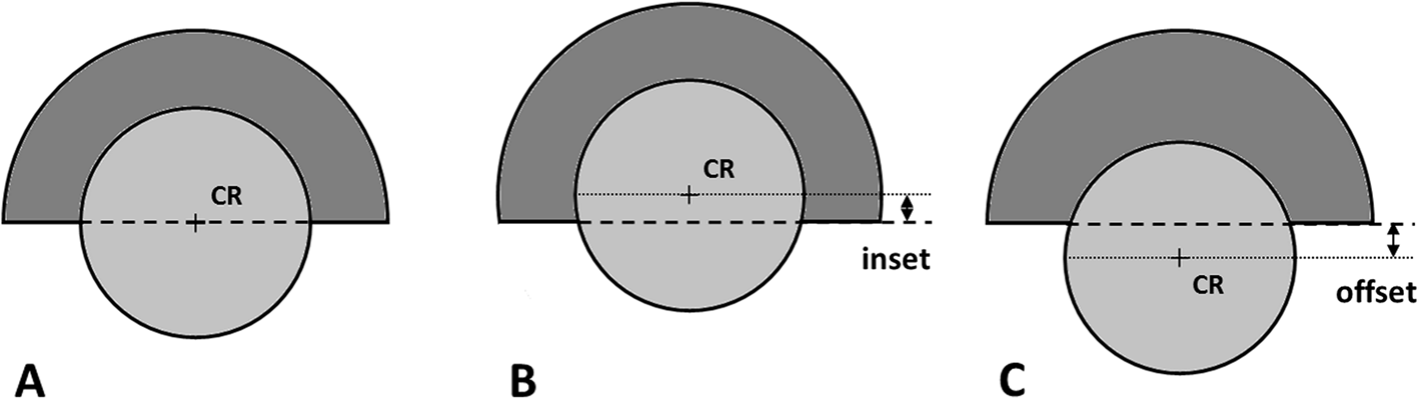
Offset of the femoral head is defined as the distance between the femoral head center (CR) and the cup opening plane. Femoral head offset: neutral (**A**), negative or inset (**B**) and positive (**C**)

*Offset* is the femoral head offset and it is defined as the distance between the femoral head center (CR) and the cup opening plane. If the femoral head center is located inside the cup, the offset has negative value (femoral inset), whereas, if it is located outside the cup, the offset has positive value (Fig. 2).

Knowing from Sariali that JD changes in function of cup abduction and anteversion angles, we decided to set the acetabular cup orientation with constant abduction angle (α) of 45° and constant anteversion angle (β) of 15°, which are within the safe zone described by Lewinnek et al. [18].

Lateralization or medialization of the CR were defined as lateral or medial shift of the CR on frontal plane when using modDM or FB in comparison with DM, as reference.

Another implant-related factor for hip stability is the prosthetic impingement-free arc of movement, or oscillation angle (OA), which is determined by the head-neck dimensional ratio and by the cup and neck specific designs. OA is defined as the maximum arc of the femoral neck axis movement within the cup, limited by the prosthetic impingement between neck and cup edge [19]. OA, as well as cup and femoral stem orientation, limited the theoretical hip range of motion (ROM).

### Prosthetic implants

All acetabular components used for this study were from the same manufacturer (Permedica Orthopaedics S.p.A., Merate, Italy), in order to exclude product design variability between different brands from different manufacturers.

We studied two cementless press-fit acetabular cups which both featured a highly-porous random trabecular titanium structure, commercially named Traser®, manufactured by selective laser melting technology without solution of continuity on the bone-implant side of the cup.

The first implant was a conventional DM cup, named Acorn Traser® DM cup, with polar-flatted hemispherical profile, 0° cup opening plane and 2.5 mm cylindrical equatorial extra-coverage. The CR of the femoral head had a medial eccentricity from the center of the polyethylene mobile liner.

The second implant was a standard titanium alloy press-fit modular cup, named Jump System Traser® cup, with a polar-flatted hemispherical profile which allowed for polyethylene or ceramic FB to be coupled with Ø28mm, 32 mm, 36 mm and 40 mm femoral heads or for modDM inlay to be articulated with the same DM liner of the Acorn Traser® DM cup.

### Methods

For measuring the distances of interest, the following landmarks were considered:

A: Center of DM liner.
C: Center of femoral head (CR).
D: Center of the ideal spherical cup outer profile.
E: intersection point between the cup opening plane and cup axis.

**Fig. 3:**
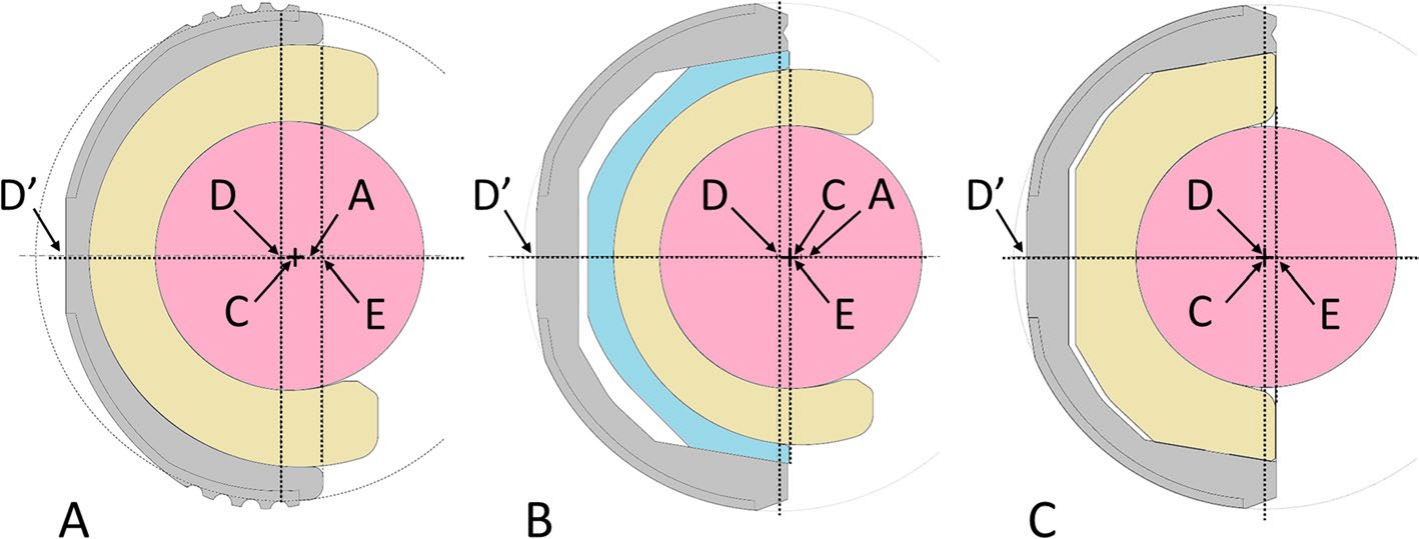
**A**, DM cup. Cross-sectional drawing of Acorn Traser® DM cup by Permedica S.p.A. **B**, ModDM system. Cross-sectional drawing of Jump System Traser® cup with modDM liner by Permedica S.p.A. **C**, FB cup. Cross-sectional drawing of Jump System Traser® cup with FB liner. Dashed lines represent the cup opening plane and the ideal spherical profile outlining the external cup profile in the press-fit equatorial area. A: Center of the polyethylene mobile liner (center of rotation of the mobile liner). C: Center of femoral head (CR of the femoral head). D: Center of the ideal spherical cup outer profile. D’: point of the cup polar apex, corresponding to the intersection between the outer cup profile and the cup axis. E: intersection point between the cup opening plane and cup axis

Offset was defined as the distance AE in case of DM and modDM, while as the distance CE in case of FB. Due to the medial eccentricity of the femoral head CR with DM and modDM, we used the center of the polyethylene mobile liner (A) as offset landmark, because when dislocating from the shell, the mobile liner acts like femoral head. R was the radius of the polyethylene mobile liner in case of DM and modDM, while the radius of the femoral head in case of FB (Fig. 3).

To calculate the CR position change, it was measured the distance CD between the CR (C) and the geometric center (D) of the ideal spherical profile which better outline the outer profile of cup equatorial portion (Fig. 3). This our convention was taken to choose a common landmark of the acetabular implant, in order to exclude design differences between DM cup and standard cup. The geometric center (D) of the ideal spherical cup profile, in fact should correspond to the center of the cup when achieving press-fit fixation into the acetabular cavity, leaving a more or less pronounced polar gap between the acetabular floor and the polar apex of the cup (D’). Thus, this center (D) approximately corresponds to the same landmark point referred to the acetabulum when implanting same-sized acetabular cups of different design. CD is then adjusted taking into account the cup frontal abduction angle Ψ, multiplying by cosΨ.

Technical 3D-models of the prosthetic components were used to simulate three configurations of acetabular implants: DM, modDM and FB, matched for same cup size (external diameter) in the range from size Ø48mm to size Ø64mm, which allowed the use of a 28 mm femoral head diameter (Table 1).

**Table 1:** Dimensional comparison of the selected prosthetic components, matched for same cup size

Cup size (external cup Ø) [mm]	Ext-Int Ø of PE mobile liner to be used with Acorn Traser® DM cup [mm]	Ext-Int Ø of PE mobile liner to be used with Jump System Traser® cup and modDM liner [mm]	modDM metal liner size (colour code)	Femoral head Ø compatible with Jump System Traser® and FB liner [mm]
48	40–28	38–28	48–50 (yellow)	28, 32
50	42–28	38–28	48–50 (yellow)	28, 32
52	44–28	40–28	52–54 (grey)	28, 32, 36
54	46–28	40–28	52–54 (grey)	28, 32, 36
56	48–28	44–28	56–60 (blue)	28, 32, 36, 40
58	50–28	44–28	56–60 (blue)	28, 32, 36, 40
60	52–28	44–28	56–60 (blue)	28, 32, 36, 40
62	54–28	46–28	62–64 (red)	28, 32, 36, 40
64	56–28	46–28	62–64 (red)	28, 32, 36, 40

In particular, the DM cup was graphically coupled with DM liner and Ø28mm femoral head and then offset AE and distance CD were measured (Fig. 3A).

Similarly, the standard cup was graphically coupled with modular DM liner, DM liner and Ø28mm femoral head and offset AE and distance CD were then measured (Fig. 3B).

Last, the same standard cup was graphically coupled with 0° polyethylene or ceramic FB and different femoral head diameters according to the available matching (Table 1) and offset CE and distance CD were measured (Fig. 3C).

Lateralization or medialization of the CR when using modDM (or FB) were defined as the difference between CD with modDM (or with FB) and CD with DM.

For OA calculation, we used, as worst case scenario, the same smallest-sized femoral stem design with a conical, round cross-sectional neck, assembled together with small neck-size femoral head, articulating with each type of acetabular component. We assumed a flat symmetrical equatorial cup edge and an implant-to-implant impingement. At the same cup position, set by α and β angles, flexion–extension, abduction–adduction and internal–external rotation OAs and total ROM were also measured for the smallest, largest and middle sizes of each considered implant.

Graphical implant simulations were performed and distances of interest were measured by using modeling and drafting tools of software CAD NX Siemens 7.5, 2010.

## Results

### Jumping distance

AE distance in DM changed from -2.4 mm with smallest cup size to -1.9 mm with largest size. Thus, the center of the DM liner (A) was located always within the DM shell, medially from the cup opening plane, so DM shell had a slightly decreasing inset as size increased (Table 2).

**Table 2:** Offset results for DM, modDM and FB with compatible femoral head diameters matched for same cup size

Cup size	DM	DM	modDM	modDM	FB	FB	FB
Ext. ∅ [mm]	Poly mobile liner ext. ∅ [mm]	Offset AE [mm]	Poly mobile liner ext. ∅ [mm]	Offset AE [mm]	Femoral head ∅ [mm]	Offset CE with polyethylene liner [mm]	Offset CE with ceramic liner [mm]
48	40	-2.4	38	1.0	28; 32	-1; -1	-1; -1
50	42	-2.4	38	1.1	28; 32	-1; -1	-1; -1
52	44	-2.3	40	1.2	28; 32; 36	-1; -1; -1	-1; -1; -1
54	46	-2.3	40	1.2	28; 32; 36	-1; -1; -1	-1; -1; -1
56	48	-2.3	44	2.0	28; 32; 36; 40	-1; -1; -1; -1	-1; -1; 0; 2
58	50	-2.4	44	2.0	28; 32; 36; 40	-1; -1; -1; -1	-1; -1; 0; 2
60	52	-2.4	44	2.0	28; 32; 36; 40	-1; -1; -1; -1	-1; -1; 0; 2
62	54	-2.2	46	3.0	28; 32; 36; 40	-1; -1; -1; -1	-1; -1; 0; 2
64	56	-1.9	46	3.0	28; 32; 36; 40	-1; -1; -1; -1	-1; -1; 0; 2

**Fig. 4:**
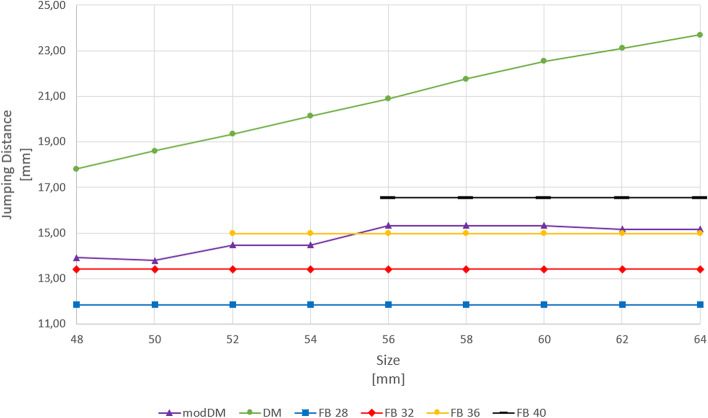
JD for DM, modDM and polyethylene FB coupled with 28 mm, 32 mm, 36 mm, 40 mm femoral head diameters per cup size

The resulting JD with DM linearly increased as size increased from 17.8 mm to 23.7 mm (Fig. 4).

AE distance in modDM changed from + 1.0 mm with smallest cup size to + 3.0 mm with largest size. Thus, the center of the DM liner (A) was located outside the shell, laterally, from the cup opening plane, so modDM cup showed an increasing offset as size increased (Table 2). JD with modDM slightly increased from 13.9 mm to 15.3 mm up to 56 mm cup size, then remained approximately constant to 15.1 mm (Fig. 4).

CE distance in polyethylene FB was set constant to -1 mm as being design parameter for all femoral head diameters (Table 2). JDs with polyethylene FB coupled with 28 mm, 32 mm, 36 mm and 40 mm femoral head diameters resulted 11.8 mm, 13.4 mm, 15.0 mm and 16.6 mm respectively, constantly per size (Fig. 4).

**Fig. 5:**
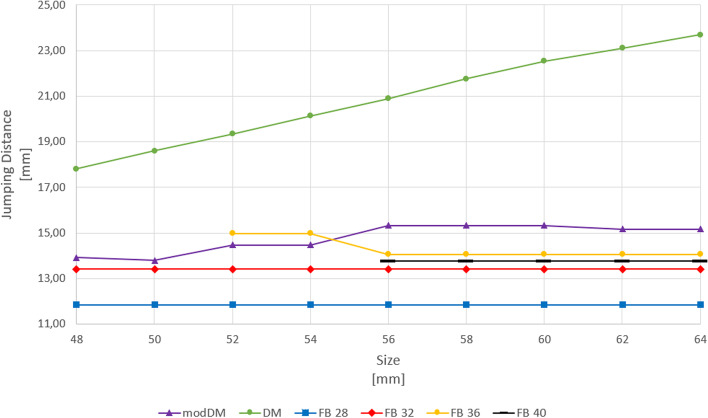
JD for DM, modDM and ceramic FB coupled with 28 mm, 32 mm, 36 mm, 40 mm femoral head diameters per cup size

CE distance measured for ceramic FB cup were -1 mm for Ø28mm and 32 mm femoral heads, 0 mm for Ø36mm and + 2 mm for Ø40mm, as constant design parameter (Table 2). JDs with ceramic FB coupled with 28 mm, 32 mm, 36 mm and 40 mm femoral head diameters resulted 11.8 mm, 13.4 mm, 14.0 mm and 13.7 mm, respectively, constantly per size (Fig. 5).

JD with modDM was reduced by -3.9 mm to -8.6 mm in comparison with DM cup, from 48 to 64 mm cup size. JD with modDM resulted comparable to JD with polyethylene FB cup with 36 mm femoral head diameter for cup sizes > 54 mm. However, JD with modDM slightly increased by + 1.1 mm and + 1.4 mm than JD with ceramic FB cup with 36 mm and 40 mm femoral head diameters for cup sizes > 54 mm.

### CD distance and CR position

**Fig. 6:**
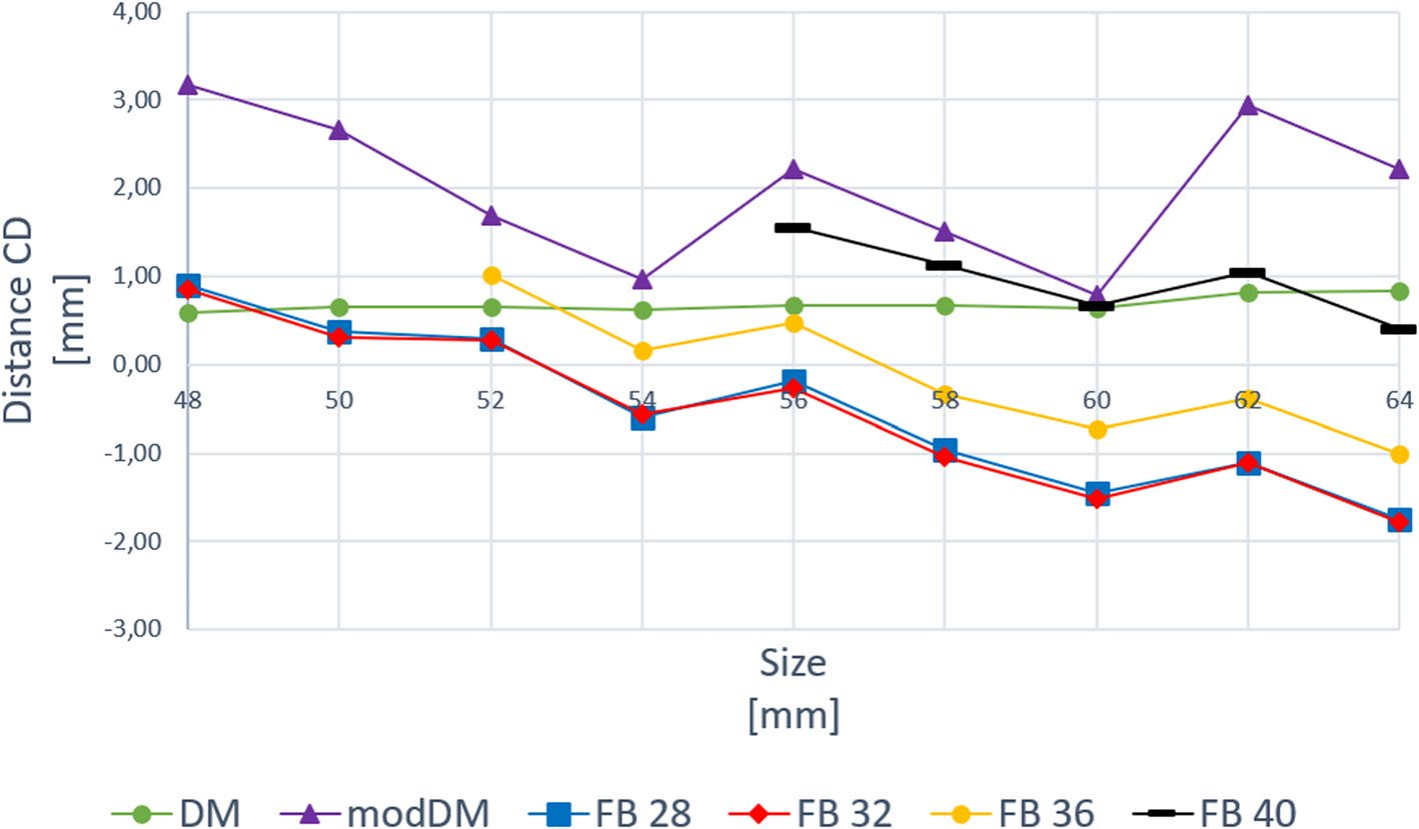
CD distance for DM, modDM and FB cups, per cup size. CD distance for FB with 36 mm and 40 mm femoral head diameters were only for ceramic liners

CD distances for DM, modDM and FB with all femoral head diameters were showed per cup size increase in Fig. 6. CD with DM slightly increased from + 0.5 mm to + 0.8 mm. CD with modDM periodically changed from + 3.1 mm to + 0.7 mm. CD with FB coupled with Ø28mm and Ø32mm heads decreased from + 0.9 mm to -1.7 mm, for both polyethylene and ceramic liners. CD with Ø36mm and Ø40mm heads coupled with polyethylene liners showed a similar decreasing trend. CD with ceramic FB and Ø36mm head decreased from + 1.0 mm to -1.0 mm, while CD with ceramic FB and Ø40mm head decreased from + 1.5 mm to + 0.4 mm.

Use of modDM involved a lateralization of the CR which ranged from + 0.1 up to + 2.5 mm depending on size in comparison with DM cup (Fig. 6). Again, modDM led to a further lateralization of the CR in comparison with FB cup, ranging from + 1.4 mm up to + 4.0 mm depending on size. Lateralization of CR with modDM even occurred in comparison with 36 mm and 40 mm femoral head diameters (Fig. 6).

### Oscillation angle

**Fig. 7:**
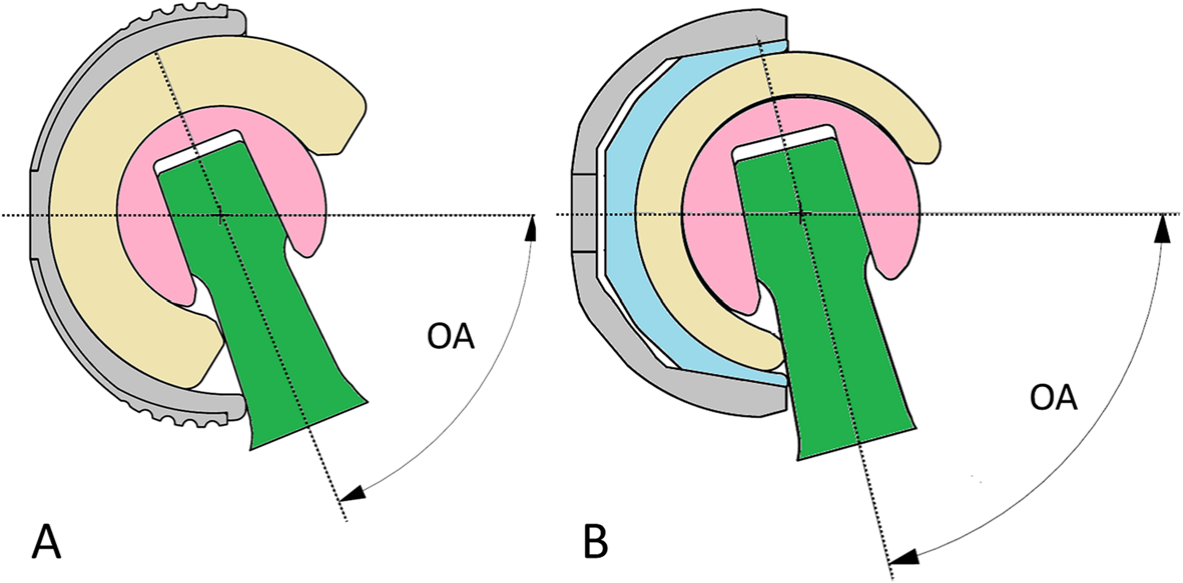
Graphical representation of the oscillation angle (OA) in modDM and DM cups

OA values and total ROM are reported in Table 3. ModDM increased OA and total ROM than DM (Fig. 7) and FB cups with 28 mm, 32 mm and 36 mm heads for each cup size. OA with modDM resulted higher than + 16°, + 23°, + 17° and + 14° in comparison to DM, 28 mm, 32 mm and 36 mm FB cups, respectively, for 56 mm cup size. Total ROM with modDM resulted higher than + 68°, + 117°, + 92° and + 74° in comparison to DM, 28 mm, 32 mm and 36 mm FB cups, respectively, for 56 mm cup size.

**Table 3:** OA, flexion–extension, abduction–adduction, internal–external rotation OAs and total ROM for the smallest, largest and middle sizes of each considered implant. NA, not applicable

	Cup size ∅ mm	DM	Mod DM	FB ∅ 28 mm	FB ∅ 32 mm	FB ∅ 36 mm	FB ∅ 40 mm
OA (°)	48	128	152	129	135	NA	NA
	56	136	152	129	135	138	156
	64	140	157	129	135	138	156
Flex-Ext (°)	48	226	270	211	222	NA	NA
	56	239	267	211	222	231	279
	64	248	285	211	222	231	275
Abd-Add (°)	48	132	151	128	134	NA	NA
	56	138	151	128	134	137	154
	64	141	157	128	134	137	152
Int-Ext (°)	48	180	218	180	188	NA	NA
	56	191	218	180	188	194	223
	64	196	226	180	188	194	218
Tot ROM (°)	48	538	639	519	544	NA	NA
	56	568	636	519	544	562	656
	64	585	668	519	544	562	645

## Discussion

After THA, hips with less JD are theoretically more susceptible to dislocate than hips with more JD. Our findings confirmed that conventional DM cups provide better joint stability achieving higher JD in comparison with modDM systems, at same cup size and same cup position. JD with DM linearly increased with increasing of cup size, similarly to the results reported by Sariali [17]. Conversely, JD with modDM resulted increasingly lower in comparison of DM as size increased.

Thus, using larger DM cups, it is possible to guarantee higher JD, so, better stability. However, this finding is not replicable with modDM which keeps JD constantly lower, whatever size of cup is used. The reason could be found looking at the formula reported by Sariali [17].

The equation highlights that JD depends not only on femoral head size, but also on cup orientation and femoral head offset. JD is mainly affected by the cup abduction angle than anteversion angle [17, 20]. With constant cup abduction and anteversion angles, JD is directly related to femoral head size and inversely related to head offset, which is the design factor that has more influence on JD [17]. Offset can have positive or negative value (inset). In case of negative sign, as with DM, an inset increase, in absolute value, leads to JD increase. Conversely, in case of positive sign, as with modDM, an offset increase leads to JD decrease and, no matter how R increases, JD remains low. So, in our simulation, offset progressively increased per cup size increase with modDM and keep JD low in comparison with DM where, instead, offset progressively decreased but remaining always negative (inset), thus increasing JD.

Regarding to polyethylene FB cup, JD changed according to Sariali [17], increasing with femoral head size increase, because femoral head offset was set constant for each size as being a design parameter (Table 2). JD with DM resulted higher than FBs of all femoral head diameters per each cup size. Interestingly, JD with modDM resulted comparable to JD with polyethylene 36 mm FB cup (Fig. 4) and JD with ceramic 36 mm and 40 mm FBs slightly decreased for sizes ≥ Ø56mm in comparison with modDM (Fig. 5). The use of large heads requires an offset increase that reduces JD [20]. For a 1-mm increase in head offset, JD is decreased by 0.92 mm. This is why very large femoral heads lead to moderate JD increase than expected and this could be an implant-related factor that could have contributed for high dislocation rates reported in revision THA with femoral heads larger than 36-mm diameter [21]. Recently, Hartzler et al. found a lower rate of hips which dislocated postoperatively after revision THA when revised with a modDM construct compared to those revised with 40-mm femoral head [22].

Our findings showed how modDM system leads to a lateral shift of the CR in comparison with DM cup and the CR lateralization amount is depending on cup and modDM liner sizes and their size matching.

All OAs and tot ROM were found higher for modDM in comparison to DM and FB implants. This finding was clearly due to both offset and lateralization of the CR with modDM and its specific design without extra-cylindrical lip which lead to a line-to-line alignment between the equatorial edges of the modDM liner and the cup, thus, allowing greater arc of movements of the femoral neck. OA is a design-dependent parameter. In fact, comparing our results with prosthetic ROMs reported by Heffernan et al. [16], it is clear that prosthetic ROMs with MDM™ were not higher than ADM™, as in the present study, because of the extra 2.4 mm cylindrical lip of the MDM™ liner.

The findings from this analytical simulation reflected the JD results from a previous simulation study with modDM (MDM™) and DM (ADM™) implants by Stryker Orthopaedics [16]. ModDM implants reduce JD in comparison to conventional DM cups and the amount of reduction is related to the specific designs of these implants. Some commercially available modDM liner designs have an extended circumferential lip to increase their JD, however this extra-coverage may reduce at the same time the OA of the stem neck.

Thus, preferring modDM implants as first choice in certain high-risk patients might unexpectedly not guarantee the same stability of conventional DM cups.

Moreover, our findings showed somewhat unexpected result that could have a clinically significant implication. ModDM resulted comparable to polyethylene 36 mm FB in terms of JD, but with a lateralized CR. In this situation, there would be no strong reasons to prefer modDM instead of a 36 mm polyethylene FB other than an increased ROM, keeping in mind that modDM might add more potential risks related to malseating and metal release.

To date, the use of modDM provided excellent results in terms of dislocation incidence. A large matched cohort single-center study comparing modDM and standard DM reported for both groups 0% of dislocation after primary THA at a mean follow-up of 2.8 years [11].

A retrospective case-series study of modDM cups used in revision THA found a dislocation prevalence of 3.1% after 3-year average follow-up [23]. Another recent multicenter retrospective study reported a similar dislocation rate (2.9%) after revision THA in a large cohort of patients treated with modDM [12].

The use of modDM is not risk-free but, conversely, involves more potential complications than conventional DM; modDM is a prosthetic construct which adds one more modular cobalt-chromium liner. The fretting and crevice corrosion processes at the non-articulating metal-on-metal interface between the modular liner and the titanium socket cause an extra metal release in comparison with conventional DM [24–26].

In literature several studies reported uniformly low blood metal ions concentrations in patients undergone modDM primary or revision THA, which were found to be acceptable for the safety of patients [13–15]. However, all these studies reported short follow-ups and the possible long-term adverse biological effects of metal release are still unknown to date. Metal release from metal modularity thus still remain a cause for concern that need to be continuously surveilled.

The use of modDM implicates also the risk of modular metal liner malseating which is reported with an incidence up to 5.8%. Liner malseating may lead to increased fretting corrosion and metal related issues, component dissociation and reduced stability [27, 28].

Thus, the use of modDM should be indicated in complex primary THA and revision THA and should be limited to those high-risk patients when the use of conventional DM cups is not recommended or even not feasible. Typical conditions that should require modDM are severe hip dysplasia, high hip dislocation, patients at high risk of dislocation with poor pelvic bone quality that requires a further cup stabilization with additional fixation screws, or revision THA for recurrent instability in case of a well-osseointegrated cup.

Facing these considerations, modDM should not be used as first choice instead of a conventional DM but rather when required or when modDM can intraoperatively solve a complication.

### Study limitations

The major limitation of the present study was that the analyses performed were strictly dependent on the design technical specifications of the studied prosthetic components. Even if the take-home message from the present study is suitable for DM and modDM THA, the authors did not exclude design-related differences between different DM cups and modDM systems currently available on the market. Therefore, the results from the present study were valid for the studied components but may change with other devices.

The focus of this investigation was on hemispherical or cylindrical-extended hemispherical cups which are both designed for standard and DM THA. In the past, JD changes were studied according to femoral head offset, head size and cup position [17]. However, these studies evaluated only standard implants and sub-hemispherical cups for large head diameters (above 38 mm), specifically designed for metal-on-metal implants, which have a negative effect on JD, due to their smaller coverage angle and higher head offset [29, 30].

## Conclusions

The findings from this study suggest that, according to the implant-specific design, hip stability after THA might be reduced in terms of JD with the use of modDM systems than conventional DM cups, resulting comparable to cups with 36 mm and 32 mm polyethylene FBs, respectively for larger and smaller cup sizes. Moreover, modDM systems might lateralize the femoral head CR in comparison to both conventional DM and FB cups, but increasing at the same time the prosthetic arc of movement. As not restoring stability parameters in the same fashion, modDM implants should be properly used in patients with high risk of dislocation for complex primary THA and revision THA, when the use of conventional DM cups is not feasible.

## Data Availability

All data are available by contacting the corresponding author.

## Abbreviations

THA: Total hip arthroplasty
DM: Dual mobility
JD: Jumping distance
modDM: Modular dual mobility
CR: Center of rotation
FB: Fixed bearings
OA: Oscillation angle
